# Clinical Epidemiology Facing New Challenges: Multimorbidity, Digital Surveillance, and Adolescent Mental Health in the Post-Pandemic Era

**DOI:** 10.3390/jcm14217498

**Published:** 2025-10-23

**Authors:** Francisco Guillen-Grima, Maria Morales-Suarez-Varela, Agustin Llopis-Gonzalez

**Affiliations:** 1 Department of Preventive Medicine, Clinica Universidad de Navarra, University of Navarra, 31008 Pamplona, Spain; 2 Navarra Medical Research Institute (IdiSNA), 31008 Pamplona, Spain; 3 Center for Biomedical Research Network Epidemiology and Public Health (CIBERESP), 28029 Madrid, Spain; maria.m.morales@uv.es (M.M.-S.-V.); agustin.llopis@uv.es (A.L.-G.); 4 Department of Health Sciences, Public University of Navarra, 31008 Pamplona, Spain; 5 Research Group in Social and Nutritional Epidemiology, Pharmacoepidemiology and Public Health, Department of Preventive Medicine and Public Health, Food Sciences, Toxicology and Forensic Medicine, Faculty of Pharmacy, Universitat de València, Av. Vicent Andrés Estelles s/n, 46100 Burjassot, Spain; 6 Department of Preventive Medicine and Public Health, Food Science, Toxicology and Forensic Medicine, Universitat de Valencia, 46100 Burjassot, Spain

## 1. Introduction

Clinical epidemiology has traditionally served as the bridge between population-based research and medical practice, offering conceptual and methodological frameworks to understand and anticipate health and disease phenomena within clinical contexts. However, in recent years, a series of transformations have begun to redefine the scope and priorities of this discipline. On the one hand, the development of new digital and analytical technologies has opened unprecedented opportunities for disease surveillance and prediction. On the other hand, the growing burden of chronic diseases and the emergence of new “silent epidemics,” such as the youth mental health crisis, demand more integrative, cross-cutting, and preventive approaches.

This Special Issue provides concrete examples of these advances, presenting research that illustrates the potential of emerging tools and innovative approaches to enhance prevention, surveillance, and disease control for both infectious and chronic conditions. This editorial aims to situate these findings within a broader context, reflecting on their relevance for the future of clinical epidemiology and public health.

## 2. Digital Surveillance: New Opportunities for Infectious Diseases

One of the most significant examples of the current transformation in clinical epidemiology is the use of digital data for disease surveillance. The study by Shih et al. explores how search patterns on Google Trends correlate with the incidence of influenza-like illnesses (ILI), providing a complementary resource for the early detection of outbreaks [1]. Through statistical and artificial intelligence models (multiple regression, ARIMA, and LSTM), the authors demonstrate that searches related to symptoms such as “fever” and “cough” can anticipate peaks in reported clinical cases.

Such approaches align with recommendations from organizations like the World Health Organization (WHO) and the European Centre for Disease Prevention and Control (ECDC), which advocate for integrating non-traditional data sources, including social networks and internet searches, to strengthen early warning systems [2,3]. The ability to detect changes in population behavior before they translate into direct healthcare demand offers clear advantages in terms of resource planning and public health response.

Nevertheless, it is essential that these tools complement rather than replace traditional surveillance systems based on clinical and microbiological data. Their integration must be guided by robust ethical and methodological frameworks that ensure the reliability and proper interpretation of information.

## 3. Multimorbidity as an Emerging Challenge in Clinical Epidemiology

The second area highlighted in this Special Issue concerns multimorbidity, understood as the coexistence of two or more chronic diseases in the same individual. The study by Rajovic et al., demonstrates that musculoskeletal diseases (MSDs), such as osteoarthritis and lumbar or cervical pain, constitute the most prevalent core in multimorbidity patterns identified within the Serbian population [4]. This finding reinforces the widely accepted notion that MSDs not only cause disability and functional decline but also act as catalysts for other chronic conditions, increasing healthcare burden and reducing quality of life.

Previous studies in Europe have pointed out the association between MSDs and psychosocial factors such as isolation, low educational attainment, and unemployment, all of which exacerbate health inequalities [5]. Furthermore, multimorbidity has been linked to increased healthcare utilization, higher hospitalization rates, and poorer health outcomes, especially in vulnerable populations [6].

From a clinical practice perspective, these findings necessitate a rethinking of healthcare models. It is vital to move from a disease-centered approach to one that addresses the complexity of patients with multimorbidity, integrating strategies for secondary prevention, rehabilitation, and health promotion tailored to specific social and cultural contexts.

## 4. Adolescent Mental Health in the Post-Pandemic Era: A Persistent Challenge

The COVID-19 pandemic has exposed not only the vulnerability of healthcare systems to infectious emergencies but also the long-term consequences on the mental health of younger generations. The SESSAMO project, conducted in Spain, provides robust evidence on the persistence of posttraumatic stress and its repercussions among adolescents two years after the pandemic began [7]. This study reveals clear associations between high levels of posttraumatic stress and increased risks of suicide, self-harm, eating disorders, and problematic use of digital technologies.

These findings reflect a growing public health concern, as highlighted by international organizations such as UNICEF and the WHO, which stress the need to prioritize child and adolescent mental health in health policies and agendas [8,9]. The consequences of this crisis affect not only adolescents’ current well-being but also their educational, social, and professional development, perpetuating cycles of vulnerability and exclusion.

Furthermore, the study emphasizes the interaction between mental health and emerging behavioral addictions, such as compulsive gaming and internet use. This phenomenon underscores the need for integrative approaches that consider new forms of risk associated with the digital environment and promote healthy lifestyles among young people.

## 5. Towards a More Integrated and Proactive Clinical Epidemiology

The studies compiled in this Special Issue share a common thread: the need to evolve towards a more integrated clinical epidemiology that incorporates population, behavioral, clinical, and digital data to anticipate health problems and design more effective responses.

On the one hand, digital surveillance offers new tools for early detection and management of infectious disease outbreaks, complementing traditional systems and expanding response capacity in changing contexts. On the other hand, multimorbidity calls for healthcare models that are more person-centered, recognizing the interrelationship between diseases, social factors, and lifestyles. Finally, youth mental health demands preventive strategies addressing both structural factors (poverty, violence, inequality) and individual factors, including emotional education and regulation of technology use.

These advances reinforce the notion that clinical epidemiology must adopt a predictive, preventive, and population-based approach aligned with the principles of P4 medicine (predictive, preventive, personalized, and participatory). This perspective involves integrating new methodological frameworks, collaborating closely with public health, and prioritizing equity in access and quality of care.

## 6. Implications for Clinical Practice and Public Health

The lessons drawn from these studies have immediate practical applications. In the realm of infectious diseases, incorporating digital sources into surveillance can enhance anticipation and preparedness for potential outbreaks, optimizing resources and minimizing health and social impacts.

Regarding multimorbidity, it is crucial to implement interventions based on health promotion, functional rehabilitation, and integrated disease management, avoiding fragmented care and reducing strain on healthcare systems. Early identification of risk factors and the adoption of preventive measures can delay or mitigate the onset of new comorbidities.

In mental health, clinical practice must strengthen its capacity for early detection, intervention, and follow-up, especially among adolescents exposed to recent traumas. Strategies should be multidisciplinary, involving not only healthcare professionals but also educators, families, and communities in a joint effort to restore protective environments and foster resilience.

## 7. Conclusions

Clinical epidemiology stands at a pivotal moment, called upon to address longstanding challenges through new perspectives. The burden of chronic diseases, infectious threats, and mental health crises are not novel phenomena, but their current manifestations require renewed tools, more integrative methodologies, and more ambitious policies.

This Special Issue contributes to shedding light on these issues, offering evidence that reinforces the importance of a clinical epidemiology capable of integrating diverse data sources, understanding the complexity of health-disease processes, and acting proactively to protect public health.

The future demands that clinical epidemiology maintain its central role in prevention, surveillance, and the improvement of population health, working in close collaboration with other fields of knowledge and adapting to the social, technological, and demographic changes that will define the coming decades.
